# CRISPR adaptation in *Streptococcus thermophilus* benefits from phage environmental DNA

**DOI:** 10.1128/msphere.00453-25

**Published:** 2025-09-22

**Authors:** F. R. Croteau, J. Tran, A. P. Hynes

**Affiliations:** 1 Department of Biochemistry and Biomedical sciences, Faculty of Health Sciences, McMaster University 3710, Hamilton, Ontario, Canada; 2 Department of Medicine, Faculty of Health Sciences, McMaster University 3710, Hamilton, Ontario, Canada; The University of Iowa, Iowa City, Iowa, USA

**Keywords:** CRISPR-Cas, adaptive immunity, bacteriophage, natural competence, eDNA, environmental DNA, phage resistance

## Abstract

The CRISPR-Cas system is a bacterial adaptive immune system that protects against infection by phages: viruses that infect bacteria. To develop immunity, bacteria integrate spacers—fragments of the invading nucleic acids—into their CRISPR array to serve as the basis for sequence-targeted DNA cleavage. However, upon infection, a phage quickly takes over the metabolism of the bacterium, leaving little time for the bacterium to acquire new spacers, transcribe them, and use them to cut the invading DNA. To develop CRISPR immunity, bacteria must be safely exposed to phage DNA. Phage infection releases environmental DNA (eDNA) which could be involved in the development of CRISPR immunity. Using *Streptococcus thermophilus* and phages 2972 and 858 as a model for CRISPR immunity, we show that eDNA is involved in CRISPR immunity, as generation of phage-immune bacterial colonies decreases with eDNA digestion. Furthermore, it is phage eDNA specifically that impacts CRISPR immunity since only its addition increases the generation of phage-immune colonies. We also show that the effect of eDNA is phage-specific, sequence-specific, and can even be traced to a region of the genome covering the early-expressed genes, which differ between phages 2972 and 858. However, we also show that eDNA is not used as a source of genetic information for spacer acquisition. These results link eDNA to the CRISPR-Cas system, providing a better understanding of the context of the emergence of CRISPR immunity and could inform our understanding of the mechanisms through which bacteria detect phage infection.

**IMPORTANCE:**

How can a bacterial adaptive immune system (the CRISPR-Cas system) exist at all, when exposure to a virulent phage is so consistently lethal? We proposed that bacteria might actively sample their genetic environment for phage DNA through natural competence. In testing this hypothesis, we revealed that free phage DNA is important to CRISPR immunity—but not as the source of the immunological memory.

## INTRODUCTION

The CRISPR-Cas system, composed of an array of clustered regularly interspaced short palindromic repeats (CRISPR) and associated *Cas* genes, is a bacterial adaptive immune system that protects against foreign DNA, including bacteriophages (1). The array is interspaced with sequences called spacers, matching invading genetic elements such as phages or plasmids (2). These spacers form the basis of the immunity as they are transcribed and processed into crRNAs (3), which then guide endonucleases to induce site-specific cleavage of any matching invading sequences (4). The development of new CRISPR-based immunity, through the acquisition of new spacers, has been observed both in laboratory and natural settings (1). Phage DNA is indubitably a source of spacer sequences, and while advances have been made in the identification of many of the molecular steps of spacer acquisition (5–12), none satisfactorily explain the “paradox of timing” inherent in this immunity.

The paradox is conceptually simple: the CRISPR-Cas system requires exposure to phage DNA to provide immunity. In the absence of resistance, intracellular exposure to phage DNA will almost invariably lead to phage replication and the death of the cell. Even transfection of purified or synthetic phage genomes into a bacterial cell can lead to production of phage particles (13). To combat this, the CRISPR-Cas system rapidly cleaves matching invading DNA, with some cleaved DNA being detected as quickly as 2 min after infection, by using pre-existing complexes of guide RNAs and Cas endonucleases (3, 14–16). The short time frame of this response is further demonstrated by the speed of the phage anti-CRISPR response. While phage tools to block CRISPRs range from dedicated anti-CRISPR proteins (17) to nucleus-like protective compartments (18), they are active within minutes of infection (19), or, in the case of nucleus-like structures, concurrently with DNA entry. Given the short time scale on which the battle for the fate of the cell is fought: how are CRISPR-naïve cells meant to acquire a new spacer, transcribe it into RNA, mature the crRNAs and then find and cleave the invading sequences before the phage has hijacked or irreparably damaged the cell?

Despite this timing issue, the CRISPR-Cas system manages to acquire new spacers from foreign sources of DNA—even in the absence of other involved mechanisms of resistance, models of CRISPR adaptation, such as *Pseudomonas* and *Streptococcus* regularly yield CRISPR-immune survivors of phage challenges at rates of ~1/10^6^ (20). These rates can be improved by synergy with other defense mechanisms (21–24) or by pre-existing partial spacer matches able to “prime” the immunity (25–27). Temperate phages capable of lysogeny, integration within the bacterial genome where they enter a state of dormancy (28), are also prime targets for CRISPR acquisition—but none of these methods directly address the problem of a naïve cell, dependent on the CRISPR system, in which such an adaptive immune system must have been selected for to defend against lytic phages. In some cases, machinery essential to the CRISPR adaptation process itself—RecBDC/AddAB complexes—degrades invading phage DNA and generate DNA free ends suitable for spacer acquisition (8). However, if this were an effective means of inactivating the phage to generate new spacers, the CRISPR system in a one-off infection would depend entirely on the success of an innate immune system.

One solution to this conundrum was demonstrated in 2014 when defective phages, capable of DNA injection but otherwise not capable of completing the rest of the infection, were shown to drive CRISPR adaptation (29). This demonstrated one way in which bacteria could be safely exposed to foreign DNA that was no longer capable of leading to cell death. This is somewhat analogous to vaccination in enabling an adaptive immune system to overcome a highly virulent virus such as rabies, where the immune system would normally have no opportunity for a repeat exposure to the virus. However, the proposed model assumed that lysates contained 10% inherently defective phages, which are responsible for >96% of all acquisition events. The authors acknowledged that this is untestable using current techniques for differentiation functional (plaque-forming units) and defective viral particles (qPCR, microscopy, particle counters). This leaves the door open for other possible ways for the bacteria to be safely exposed to phage DNA.

Bacteria are naturally exposed to foreign DNA in one of three main ways: transformation (natural competence), conjugation, and transduction. The acquisition of spacers from DNA injected by defective phages is similar to transduction, and spacers are readily acquired from plasmids (30), making it likely that conjugation could lead to acquisition. However, transformation has yet to be linked to the development of phage immunity despite the readily available phage genetic material in the environment. In the context of phage infection, phage-induced lysis will cause the release of any number of remaining intracellular components, including bacterial and phage proteins, small molecules, such as nucleotides, bacterial DNA, and unencapsidated phage DNA (31). Since the CRISPR-Cas system uses the sequence of invading phages as the basis for immunity, this phage environmental DNA (eDNA) contains the genetic information necessary for CRISPR adaptation and defense and could be taken up by naturally competent bacteria through transformation. Moreover, before being taken up, the eDNA is cut into smaller pieces (32), and one of the strands of the eDNA is degraded so that only a single strand of DNA enters the cell (33, 34). Since previous work has shown links between ssDNA and CRISPR adaptation (35) in the context of exposure to phage genetic material, this would make transformed phage DNA a potentially safe source of phage genetic information for the bacterium. We hypothesized that unencapsidated phage genomic DNA released as part of phage lysis could be involved in the development of CRISPR immunity.

Using a model of CRISPR-Cas adaptation, *Streptococcus thermophilus* DGCC7710, we show that eDNA is an important contributor to the generation of new CRISPR immunity, that this effect is specific to the phage—requiring the environmental DNA to match the challenging phage, but surprisingly does not occur by providing a source of genetic material for spacer acquisition.

## RESULTS

### Phage lysates contain both bacterial and phage eDNA

To confirm the presence of eDNA in phage lysates, we measured the concentration of both bacterial and phage eDNA using qPCR with species-specific primers in lysates of phage 2972 and phage 858. Bacterial eDNA was detected in phage 2972 lysates at 0.13 (3) ng·µL^−1^ (*n* = 9) and in phage 858 lysates at 0.157 (3) ng·µL^-1^ (*n* = 4) for an overall average of 0.14 (2) ng·µL^−1^ (*n* = 13). Uncertainty on calculated or measured values is indicated with the parenthesesr applied to the smallest significant digit. This corresponds to approximately 7(2)e7 genomes per milliliter, in keeping with cell counts at time of infection (~5e7 cfu/mL).

To detect only phage eDNA, we had to assume the high temperature (95°C) of the PCR could result in the release of packaged phage DNA. In order to calculate the concentration of phage eDNA present before qPCR, we subtracted the value of phage eDNA measured in a lysate treated with DNase, which contains eDNA released during the qPCR and during the inactivation of the DNase, from the value measured from the same lysate treated with DNase buffer only, which contains the original eDNA as well as eDNA released during both the qPCR and the inactivation of DNase. Phage eDNA was calculated to be, in phage 2972 lysates, 0.5(1) ng·µL^−1^ (*n* = 9) and, in phage 858 lysates, 0.4(2) ng·L^-1^ (*n* = 6), for an overall average of 0.5(1) ng·µL^−1^ (*n* = 15). This corresponds to 1.3(4)e10 and 1.1(4)e10 genomes per milliliter for phages 2972 and 858, respectively. In our lyates , for each infective particle (pfu), there were ~100 phage genomes of eDNA, enough for more than 50 free-floating phage genomes per bacterial CFU in a typical bacteriophage-insensitive mutant (BIM) assay. These values seem high, but similar ratios of qPCR-to-infectious particle have been reported in the past (36), suggesting that some phages have high ratios of “wasted” genomes not packaged into infective particles. There was no significant difference in the eDNA value measured across the lysates from the two different phage species (Welch’s Anova, *P* > 0.05). We also plotted the calculated eDNA concentration in each lysate as a function of phage titer and determined through linear regression analysis that there was no significant correlation between phage titer and phage eDNA concentration for either phage (Fig. S1).

To establish the efficacy of the DNase treatment in phage lysate, we measured bacterial eDNA concentration in DNase-treated lysates. After digestion, the average concentration of bacterial eDNA in lysates dropped to 0.00(2) ng·µL-^1^ (*n* = 13), which is a significant reduction in measurable eDNA level when compared with the untreated condition (*P* < 0.001, Welch’s ANOVA, Games-Howell test). In short, phage eDNA is present at a high level in these lysates, and DNasee digestion does effectively remove it.

### DNase treatment reduces BIM generation

**Fig 1:**
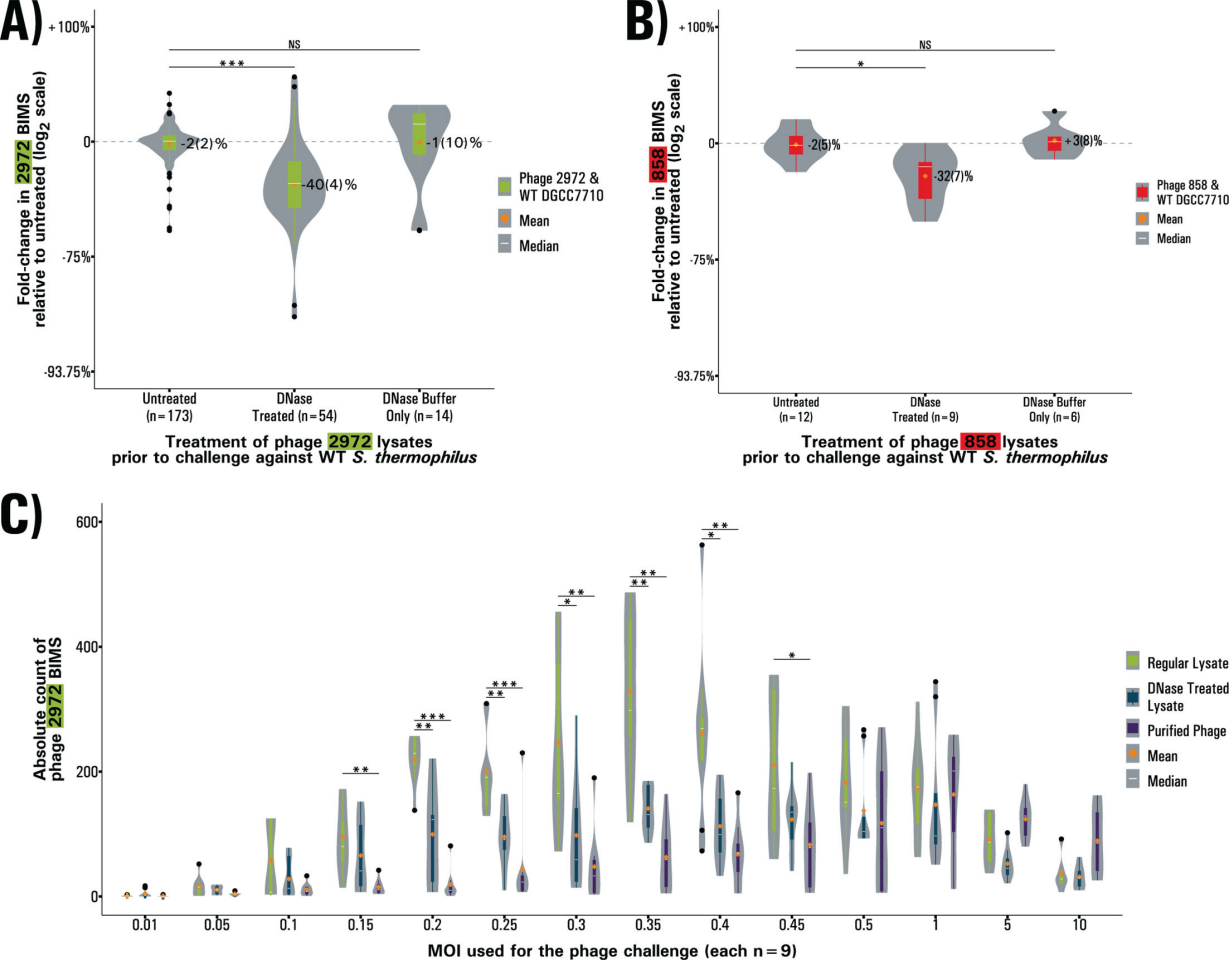
BIM generation with DNase-treated lysate and purified phage (**A and B**) BIMs obtained in challenges against phage 2972 (**A**) and phage 858 (**B**) at MOIs between 0.15 and 0.45. BIMs are reported as fold change relative to the baseline. (**C**) BIMs obtained in challenges against phage 2972 at varying MOIs. BIMs are reported as absolute counts for each condition in either untreated lysates, DNase treated lysates, or purified phages. In all cases, violin plots represent the probability density curve of the distribution, boxplots represent the first and third quartiles of the distribution (box), the minimum and maximum (whiskers), the median (white line) as well as any outliers (black dots). The mean of each distribution is represented by an orange diamond. Significance is determined by pairwise comparison with the untreated condition using Welch’s ANOVA followed by Games-Howell post-hoc pairwise comparison test.

Having confirmed the presence of phage eDNA in lysates and the efficacy of DNase in digesting it, we tested the effect of eDNA digestion on the generation of CRISPR-mediated immunity by comparing the number of bacteriophage-insensitive mutant (BIM) colonies obtained after a phage challenge with untreated or DNase-treated lysates, using lysates treated with DNase buffer only as a control. The challenges we performed at a multiplicity of infection (MOI) between 0.3 and 0.4, where BIM generation peaks (20). In all conditions, acquisition of spacers in the CR1 or CR3 arrays of nine BIMs was confirmed by PCR. All BIMs showed acquisition of at least one spacer with acquisition in CR3 happening only in a single BIM. This pattern of acquisition is consistent with what was previously observed for this strain (20, 37). In challenges with phage 2972, the DNase-treated lysates yielded 40(5)% fewer BIMs than the untreated control condition (Welch’s ANOVA, Games-Howell test, *P* < 0.001). The untreated condition produced a number of BIMs consistent with the expected average acquisition rate of ~1 in 1e6 (20). The DNase buffer condition did not result in a significant difference from the control (Fig. 1A). This experiment was repeated using phage 858 for the challenge and yielded similar results with DNase-treated lysates resulting in 32(7)% fewer BIMs than the untreated control condition (*P* < 0.05, Games-Howell test) (Fig. 1B).

We attempted to recapitulate the phenotype by digesting eDNA with various restriction enzymes. However, digestion by restriction enzymes was not sufficient to cause a significant effect on BIM generation (Fig. S2). We also verified that the DNase treatment did not affect the concentration of plaque-forming units (PFU) in phage 2972 and found that the variation in titer as calculated by full plate PFU count assays was 1(1)%, which is not significant (Tukey’s HSD test, *P* > 0.05) (Fig. S3). Since there is no effect caused by treatment with DNase buffer only, the reduction in BIMs is therefore due to the degradation of eDNA by DNase. This shows that eDNA plays a role in the acquisition of resistance against phages.

### The effect of eDNA is MOI-dependent

To verify if this effect of DNase was universal across multiple MOIs, we performed similar challenges at multiple MOIs ranging from 0.01 to 10. We performed these challenges using untreated lysates, DNase-treated lysates, as well as purified phage solutions obtained by PEG precipitation and ultracentrifugation to remove not only eDNA but also other lysate components. Since when using multiple MOIs, there is no obvious reference condition, we reported observations as absolute BIM counts as opposed to relative fold change. In untreated lysates, BIM generation peaked at a MOI of 0.35, confirming prior results indicating this MOI range as optimal for generation of CRISPR immunity (20). The DNase treatment had a significant effect on BIM generation at MOIs between 0.2 and 0.4 (*P* < 0.05, Welch’s ANOVA, Games-Howell test) (Fig. 1C). The purified phage solution yielded significantly fewer BIMs than the control at MOIs between 0.15 and 0.45 (*P* < 0.05, Welch’s ANOVA, Games-Howell test) (Fig. 1C), with a peak now at a MOI of 1. This shows that the effect of eDNA is MOI dependent, as at higher MOI, all conditions yielded similar amounts of BIMs. We suggest that, at lower MOIs, uninfected cells benefit from the “first wave” of lysis by involving the eDNA released in CRISPR adaptation, and, as the MOI increases and there are fewer uninfected cells, the importance of eDNA decreases.

To determine whether eDNA was solely responsible for this effect, we supplemented purified phage with an excess of purified phage gDNA in an attempt to restore the phenotype. However, the supplementation of phage DNA did not increase BIM generation when compared with untreated purified phage (Fig. S4). This indicates that while eDNA is necessary for “optimal” BIM generation, it is either not sufficient or differs qualitatively from the supplemented, purified DNA. The effect likely requires other cofactors to be effective. We tested if the addition of the ComS competence-inducing peptide alongside eDNA could be sufficient to restore the phenotype since this short signal peptide has been shown to induce natural competence in *S. thermophilus* (38) but the addition of the peptide alongside eDNA had no effect (Fig. S4).

### Competence is likely necessary for the effect of eDNA

In order to test if eDNA was taken up by the bacteria to provide its immunogenic effect, we generated a scarless deletion of the *comEC* gene, which encodes the ComEC channel protein—a necessary part of natural competence in Gram-positive bacteria (39). The sensitivity of the Δ*comEC* strain to both phages used was tested using an efficiency of plaquing (EOP) assay, yielding an EOP of 0.997 for phage 2972 and 1.029 for phage 858, confirming that the deletion of the *comEC* gene did not impact the sensitivity to either phage. When comparing BIMs obtained by the two strains, since OD_600_ could not be exactly matched, we reported absolute number of BIMs obtained across multiple experiments with varying OD_600_ values chosen to obtain an MOI between 0.15 and 0.45. In the Δ*comEC*, strain the average absolute number of BIMs generated was reduced by 64% when compared with the WT strain (*P* < 0.01, Welch’s analysis of variance [ANOVA], Games-Howell test) (Fig. S5A). Moreover, the effect of DNase treatment of lysates on BIM generation was lost when the Δ*comEC* strain was used for phage challenges (Fig. S5B). Due to supply constraints of the growth medium (see results on Oxoid LM17 below), we were unable to test a complementation strain to confirm these results were due only to the *comEC* deletion. However, we believe the lower BIM generation and loss of the effect of DNase in the Δ*comEC* strain indicates that eDNA is taken up through competence in order to affect CRISPR adaptation.

### Supplementation of phage eDNA can increase BIM generation in regular lysates

**Fig 2:**
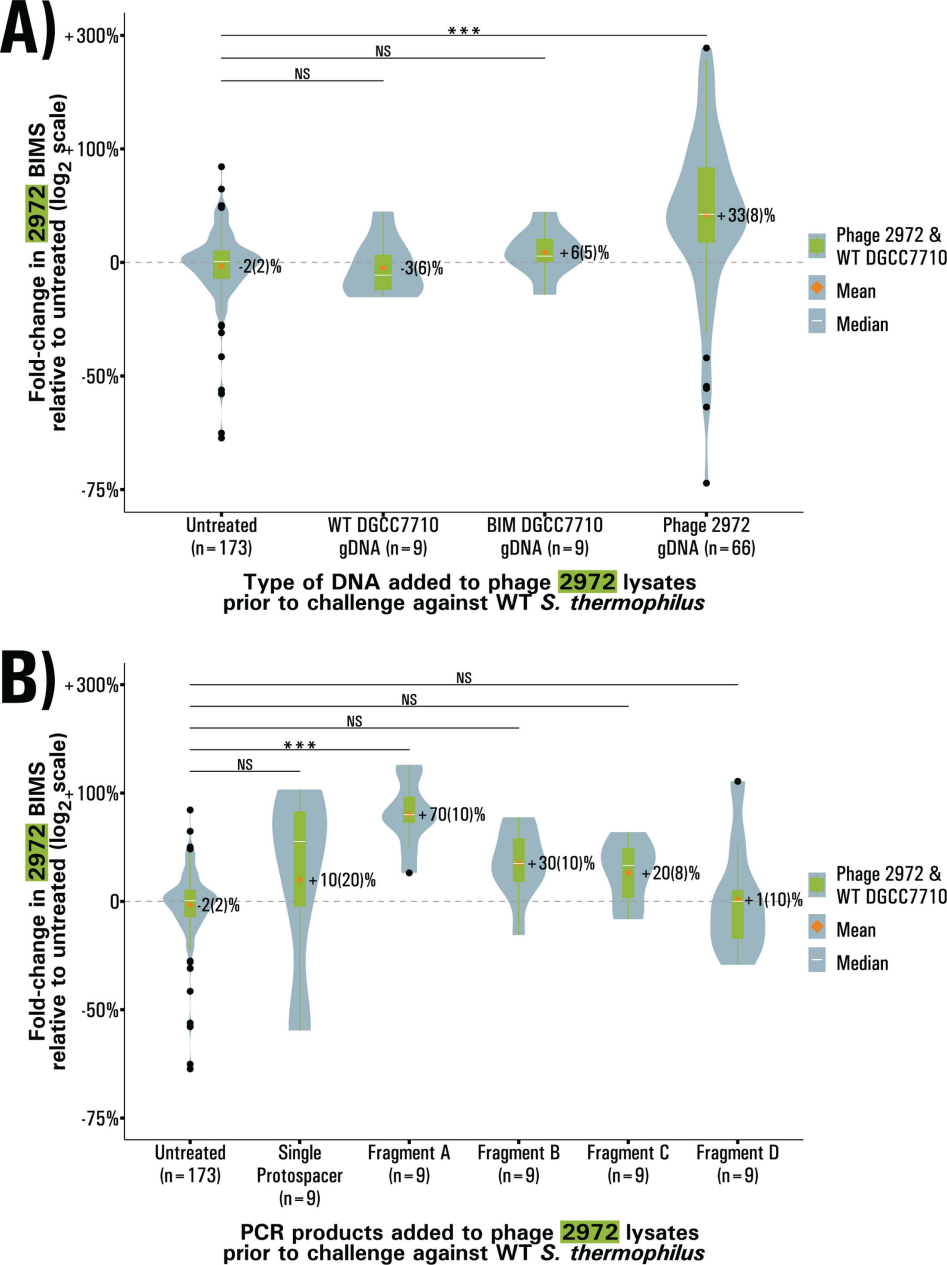
BIM generation with lysates supplemented with purified DNA. (**A**) BIMs obtained in challenges with phage 2972 lysates supplemented with 10 ng·μL^−1^ of DNA from various sources. (**B**) BIMs obtained in challenges with phage 2972 lysates supplemented with 10 ng·µL^−1^ of PCR amplicons. In all cases, BIMs are reported as fold-change relative to the baseline, violin plots represent the probability density curve of the distribution, boxplots represent the first and third quartile of the distribution (box), the minimum and maximum (whiskers), the median (white line) as well as any outliers (black dots). The mean of each distribution is represented by an orange diamond. Significance is determined by pairwise comparison with the untreated condition using Welch’s ANOVA ,followed by Games-Howell post-hoc pairwise comparison test.

In order to determine which properties of eDNA were responsible for its immunogenic phenotype, we tested if we could bolster BIM generation by supplementing purified DNA to phage lysates. Ideally, this experiment would have been performed in a DNase-treated lysate, but we were unable to inactivate or sequester the DNase without affecting the viability of infectiousness of the phages. First, we supplemented to a final concentration of 10 ng·μL^−1^ whole genomic DNA from WT *S. thermophilus*, from a BIM *S. thermophilus* strain with a spacer targeting phage 2972, from phage 2972 itself and from the related phage 858. This was to verify if the effect of eDNA was due to any DNA molecule, if bacteria could use pre-adapted CRISPR arrays to expand their own through recombination, or if phage DNA was required. Across these conditions, only the supplementation of DNA from phage 2972 resulted in a significant increase (*P* < 0.001, Welch’s ANOVA, Games-Howell test), with 33(8)% more BIMs generated (Fig. 2A). This confirms that phage DNA is driving the effect of eDNA on BIM generation. Moreover, despite sharing most of its genome with phage 2972, the supplementation of the related phage 858 DNA did not increase BIM generation upon selection with 2972, potentially due to factors unique to each phage’s genome—such as an unknown modification system. The ability of purified phage gDNA to increase BIM generation also rules out the hypothesis that phage DNA alone could not restore BIM generation to purified phage because it was qualitatively different from the eDNA found in the lysates, making the involvement of cofactor present in regular lysates even more likely. We hypothesize that this cofactor could be either released along with unpackaged DNA during lysis or potentially even secreted by bacteria as a response to their own infection by phages or the infection of neighboring cells.

### Specific sequences of phage eDNA are responsible for the increase in BIM generation

To examine the mechanism through which phage eDNA contributes to CRISPR adaptation and to determine whether epigenetic factors are responsible for lack of effect observed when supplementing DNA from the related phage 858, we designed PCR amplicons of varying sizes across the genome of phage 2972. We identified phage genomic regions containing similar number of protospacers—the regions next to the protospacer-adjacent motifs that allow for CRISPR acquisition—that could be potentially targeted by the CRISPR 1 (CR1) array of *S. thermophilus*. This array was chosen since it is responsible for upwards of 90% of all acquisition events in this system (37).

**Fig 3:**
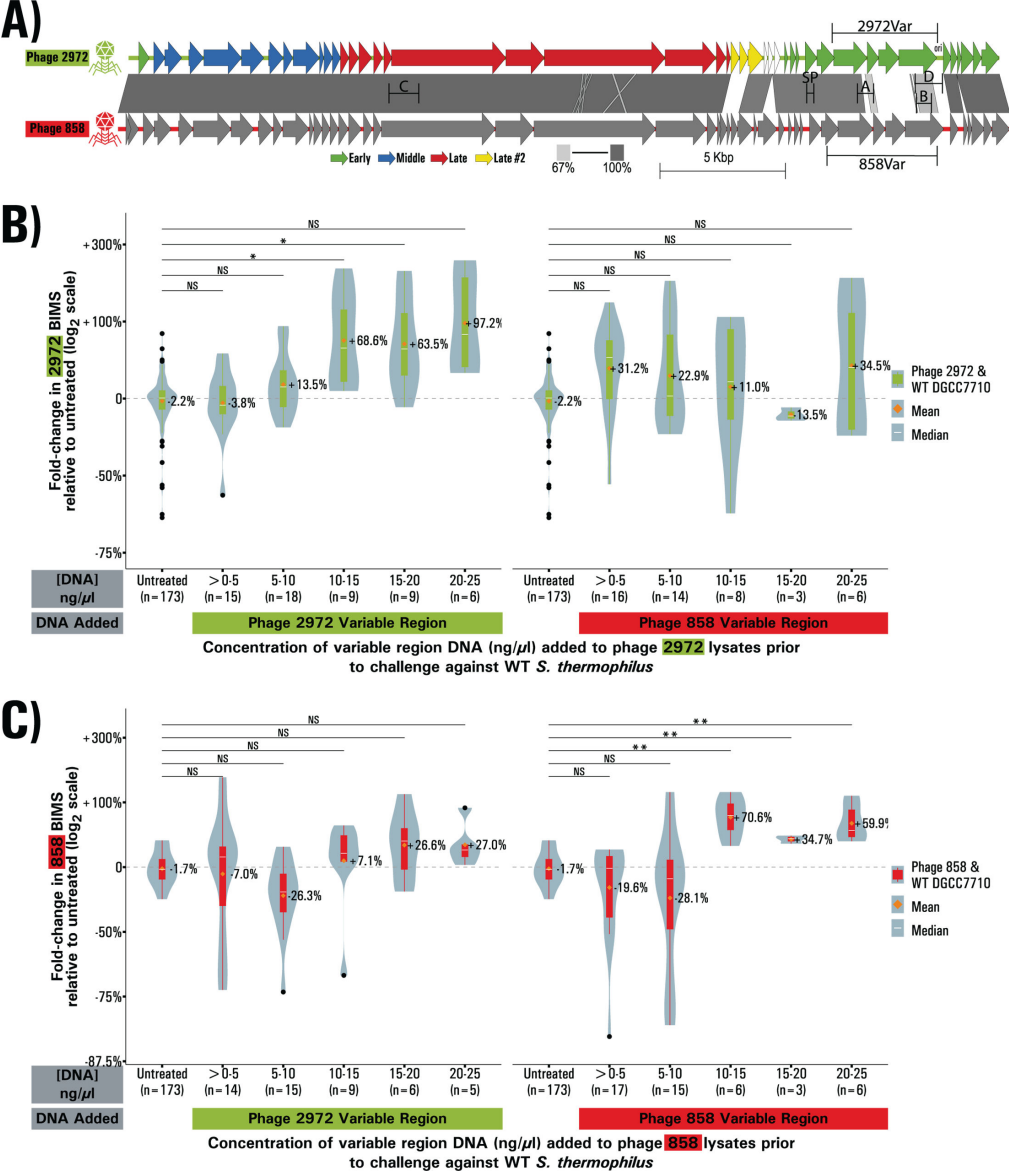
Genomic regions of variability between phages 2972 and 858 increase BIM generation. (**A**) Genomic comparison of phages 2972 and 858. CDS are shown as arrows, percent homology by blastn is shown by a gray bar between the genomes, scale from 67% to 100% homology. Transcription modules of phage 2972 according to Duplessis et al. are shown by colors (green, blue, red, and yellow for early, medium, late, and late II transcribed genes). The origin of replication for phage 2972 is shown as ori. The positions of the PCR amplicons used in various experiments are also shown using the letters for fragments A-D, SP for the single Protospacer amplicon, and 2972Var and 858Var for the two variable regions of each phage. (**B and C**) BIMs obtained in challenges with phage 2972 (**B**) and phage 858 (**C**) lysates supplemented with the variable region of both phages at varying concentrations. Conditions are grouped in bins of concentration of 5 ng/µL. The origin of the variable region is indicated by a colored bar under the X-axis. BIMs are reported as fold change relative to the baseline, violin plots represent the probability density curve of the distribution, boxplots represent the first and third quartile of the distribution (box), the minimum and maximum (whiskers), the median (white line) as well as any outliers (black dots). The mean of each distribution is represented by an orange diamond. Significance is determined by pairwise comparison with the untreated condition using Welch’s ANOVA ,followed by Games-Howell post-hoc pairwise comparison test.

We designed five amplicons; two amplicons of approximately 600 bp containing seven protospacers, two of approximately 1,000 bp containing 10–11 protospacers, and one amplicon of 282 bp containing a single protospacer (see Fig. 3A for positions). This allowed us to confirm if the length, position on the genome, or protospacer density impacted the effect of eDNA. To our surprise, only the supplementation of a single fragment, Fragment A (Fig. 2B), resulted in a significant change in BIM generation (*P* < 0.001, Welch’s ANOVA, Games-Howell test); a 70(10)% increase. This confirms that epigenetic factors such as DNA modification are not responsible for the effect of eDNA and also indicates that specific sequences are likely responsible.

### The genomic differences between phage 2972 and phage 858 are clustered

Since the PCR fragment supplementation experiments indicate that epigenetic factors are likely not involved and since only the supplementation of phage 2972 DNA was able to increase BIM generation in a phage 2972 challenge, we surmised that the sequences responsible for this effect are likely located in genomic regions of high variability between phages 2972 and 858. Alignment of the two genomes confirmed that they have 90.2% pairwise identity and indicated not only global homology but also a similar genomic organization (40). Most of the differences are clustered in a region spanning from 28,505 to 32,484 in phage 2972, which covers the ORFs 35 to 38 (phage 858 ORFs 37 to 40) (Fig. 3A). A search on NCBI’s conserved domain database showed that nearly all functional domains identified are identical between the four ORFs, indicating a likely shared function despite little pairwise identity at both the nucleotides and amino acid levels. In phage 2972, the region was previously identified as covering the early expressed genes (41), and due to the similarity in annotations between the two phages, it is likely that these genes have similar expression patterns in phage 858. Serendipitously, the fragment A PCR amplicon was located within this variable region, reinforcing the importance of this region to the effect of eDNA.

### A specific region of the phage genome is responsible for the effect of eDNA

To further examine this region, we designed PCR amplicons spanning most of the variable region for both the genomes of phage 2972 and phage 858. We then performed phage challenges using both phage 2972 and phage 858 lysates either untreated or supplemented with various concentrations of variable region PCR amplicon DNA. This was done to test whether the effect of supplemented DNA would vary with the concentration used. We saw no dose-response effect, and no linear correlation. When we binned the data in groups in order to increase the number of replicates in each group, we noticed that the increase was only significant (*P* < 0.05, Welch’s ANOVA, Games-Howell test) when DNA of the challenging phage was supplemented to a final concentration above 10 ng·µL^−1^. This post-hoc analysis explains the variability in replicate number in each bin. The increase in BIMs observed at higher concentrations of DNA was greater in amplitude than the increase caused by supplementation of whole genome DNA or PCR fragment A. In other words, only a phage’s own early region is able to cause an increase in BIM generation (Fig. 3B and C) and appears better at doing so than the whole genome.

### eDNA does not serve as a source of genetic material for spacer acquisition

To convincingly show that eDNA provides genetic information for spacer acquisition, we tracked which spacers were acquired by BIMs obtained from challenges with the lysate supplemented with PCR fragment A. Since supplementation of the fragment A resulted in an increased number of BIMs, if eDNA provides genetic information for spacer acquisition, we would expect an over-representation of spacers matching the protospacers present on the amplicon. We sequenced 16 BIMs obtained from the lysate supplemented with fragment A, out of which 12 had acquired a distinct spacer in their CR1 array. When mapping the 12 acquired spacers to the genome of phage 2972, no acquired spacer mapped to the fragment A amplicon; instead, the spacers mapped across the genome without any clear pattern.

To verify how likely this scenario was if eDNA does act as a source of protospacers, we performed a binomial distribution test composed of the following assumptions: (i) eDNA provides genetic material for new spacer acquisition, (ii) due to the 70(10)% increase in BIMs when using lysate supplemented with fragment A, 42.7% of the BIMs are due to the effect of eDNA, (iii) there is a 3% (7/233 protospacers) change of acquiring a spacer mapping to the amplicon from BIMs not caused by eDNA, and (iv) there is a 90% chance of acquisition in CR1 (37). This results in a probability of any acquired spacers to map back to fragment A of 41.1% ($90\%*\left(42.7\%+3\%\right)$). Using binomial distribution, we can establish that the probability of observing 0 spacers mapping to fragment A across 16 unique BIMs by chance alone is 2.07e-4, allowing us to confidently reject the hypothesis. Since three of our four assumptions are experimentally supported, the most likely faulty assumption is that eDNA provides genetic material for new spacer acquisition.

We arrived at the same conclusion using another experiment based on the search for the acquisition of non-protective spacers. We infected a culture of *S. thermophilus* DGCC7710 in LM17 broth with phage 2972 lysate supplemented with DNA from phage 858. While these two related phages share some protospacers which when acquired would be protective against both phages, they also contain unique protospacers that are only protective against a single phage. We targeted 10 protospacers unique to phage 858 using a multiplex PCR strategy with a shared forward primer binding upstream of the CRISPR array and reverse primers specific to each spacer. As these spacers would not be protective, we extracted the total DNA of the liquid culture (eDNA + bacterial gDNA) to use as a template for the multiplex PCR. In cultures challenged with phage 2972 lysate supplemented with phage 858 DNA, we detected no acquisition of the 10 targeted unique protospacers while they were all detected by gel electrophoresis when tested individually in the culture challenged with phage 858 used as a control. To verify how likely that scenario is, if eDNA acts as a source of protospacers, we performed a binomial distribution test composed of the following assumptions: (i) eDNA provides genetic material for new spacer acquisition, (ii) in seven 5 mL cultures at a bacterial concentration established by a growth curve as ~1e8 CFU/mL and using a CRISPR acquisition rate of ~1 in 1e6, we can expect 3,500 individual BIMs, (iii) we can expect 40% of BIMs to be due to eDNA considering the 40(4)% reduction in BIMs caused by DNase treatment, (iv) 95.2% of eDNA BIMs are due to the supplemented phage 858 DNA as we supplemented 10 ng/µL of DNA to an average phage eDNA concentration of 0.5(1) ng/µL as established earlier, (v) only 4.3% (10/234) of phage 858’s protospacers are targeted by the multiplex primer mix, and (vi) only 25% of the extracted DNA was used for the PCR reaction. This results in a probability of any acquired spacers being one of the 10 targeted spacers of 0.4% (40%*95.2%*4.3%*25%). Using the binomial distribution, we can establish that the probability of not finding any of the targeted spacers across the entire set by chance alone is 6.32e-7, which allows us to reject the hypothesis. Similarly, all but one of our assumptions is experimentally derived, and the most likely faulty assumption is that eDNA provides sequence information for CRISPR acquisition. However, this experiment assumes that the DNA extraction process is perfect, and that no DNA is lost. If we account for potential loss of DNA during extraction and poor PCR efficiency, even combined DNA retention rates as low as 22% still yield a probability of randomly not detecting any acquisition below 0.05. While a direct proof of a negative is impossible, these experiments show that eDNA is not likely to provide genetic information for spacer acquisition despite previous work showing that ssDNA can be used as part of the acquisition process (35).

### The effect of eDNA is limited to oxoid LM17

Throughout the process of performing these experiments, we used LM17 media from the Oxoid (Nepean, Canada) brand. Eventually, Oxoid brand LM17 became unavailable, and upon switching to another supplier, we realized that the effect of eDNA on CRISPR adaptation was lost in other brands of LM17. While we were able to generate BIMs using LM17 media obtained from brands Difco (Franklin Lakes, USA) and Tekniscience (Terrebonne, Canada), there was no longer a significant reduction in BIM generation caused by DNase treatment (Fig. S5A). Similarly, the effect of eDNA supplementation was also lost in LM17 from Difco and Tekniscience (Fig. S5B). In an attempt to elucidate this loss of the phenotype, we tested the stability of eDNA and the activity of DNase in LM17 from the various brands as well as in spent cultures in the same media and found that eDNA is stable and DNase is active in all media types. We also attempted to make LM17 from raw ingredients but failed to grow *S. thermophilus* to high enough OD_600_ to perform BIM assays. We are confident that this effect is due to the media and not a change in our strains since Oxoid LM17 became unavailable twice during the course of this study. The first time lasted only for a few months during which we observed experimental failures that were not investigated further when Oxoid LM17 became available again, resolving the issues. The second time Oxoid became unavailable with no clear end to the shortage, we investigated the effect of the media more deeply, gathering the data presented above.

## DISCUSSION

We showed here that eDNA plays a role in the development of immunity against phages (Fig. 1) and that it is phage eDNA matching a specific region corresponding to some of the early expressed genes that is responsible for this effect (Fig. 2 and 3). Both removal and supplementation of eDNA in phage lysate influence the number of BIMs generated, making a clear connection between the naturally present phage eDNA in phage lysate and BIM generation. In the absence of eDNA, we can reduce BIM generation by 40%, and by spiking in the right DNA, we can increase BIM generation by up to 70%—suggesting that eDNA can theoretically increase BIM generation almost threefold. While the specifics of this interaction remain unknown, we have uncovered some of the elements required for the effect of eDNA on BIM generation.

First, the loss of the effect of eDNA in a competence-deficient background strongly indicates that the eDNA is taken up by the bacteria to mediate this effect (Fig. S5). However, supplementation of eDNA does not lead to a bias in the acquisition of spacers matching the protospacers present on the supplemented sequences. This shows that while the effect of eDNA is mediated from inside the cell, it does not provide early access to genetic information for spacer acquisition.

We posit that the effect of eDNA is due to a direct sequence recognition of the eDNA to the phage genome rather than a mechanism driven by the function of the gene product encoded by the eDNA that is taken up. This is supported by the fact that the eDNA can be partially degraded, as shown by our restriction enzyme experiments (Fig. S2), only specific regions of the phage genome can affect BIM generation when supplemented, and while this effect happens in two different phages, the phage sequences targeted are not cross-reactive. If the effect were caused by the gene products, as their predicted functions are similar in both phages, we would expect a cross-reactivity when supplementing DNA from another phage species. However, further studies into the role of these proteins in phage-bacteria interactions might still be of interest, particularly if the effect is mediated by interference with expression of these proteins at the DNA or RNA level.

We were curious if the nature of the variable regions would shed light on the mechanism and searched bioinformatically for conserved genomic features, such as inverted repeats, palindromes, tandem repeats, and hits to known RNA structures from the Rfam database. While there were multiple hits across both phages, they were poor matches. Furthermore, none of these hits were located in Fragment A, which was shown to be sufficient to increase BIM generation. This makes it unlikely that these features play a mechanistic role in the observed phenomenon.

We also sought to investigate this “immunogenic” variable region across *Streptococcus* phages, searching for evidence that it might identify multiple distinctive regions, and perhaps reveal a selection to avoid cross-reactivity between phages. We analyzed genomes of at least 10,000 bp labeled as “*Streptococcus* phage” in either the title or organism field containing a host annotation for *S. thermophilus* and downloaded as such 130 genomes. Of these, 66 contained a blast (42) hit to at least one of either the 2972 variable region or the 858 variable region, and 50 contained hits in both (the Fragment A alone was also tested but resulting hits were equivalent to that of the whole 2972 variable region). With these parameters, the 2972 variable region did not match the phage 858 genome (and vice versa), but the phage genomes that matched both (albeit at varying percent identity) seem to indicate that these phages are intermediates between the two phages in sequence. In contrast, only 11 genomes matched only phage 2972, and five matched only 858. We see no evidence of any particular clustering of this region, although it could be interesting to see to what extent these partial matches would increase BIM generation when supplemented as eDNA.

We propose a model where phage eDNA is taken up by the cell—likely due to some signal released by neighboring infected cells, and some specific sequences of the phage then interfere with the typical infection cycle—either directly, or through an unknown bacterial system—allowing more opportunities for the bacteria to acquire spacers. The possibility of slowing or blocking phage DNA replication as an adjuvant mechanism of resistance is not unheard of. Previous work has shown that the presence of a phage origin of replication on a plasmid transformed into *S. thermophilus* leads to resistance to phage by preventing the accumulation of phage DNA (43). While the origin of replication of phage 2972 is outside of the variable region PCR amplicon and the Fragment A amplicon, the effect of this region on BIM generation might still be caused by a similar mechanism of stalling phage DNA replication. The early steps of DNA replication involve multiple protein-ssDNA interactions (44, 45), all of which could be impacted by competitive binding to “dummy sequences” acquired from eDNA. The work of Foley et al. also highlighted sequence specificity localized to the region of the replication modules. While their work focused on the presence of the origin of replication, it is possible that the two effects work through a similar mechanism that is colocalized with the origin of replication but does not necessarily require it. Interestingly, the loss of the importance of eDNA at high MOIs might support this theory as well, as co-infections by the same phage would result in higher copy numbers of the phage genome (and the relevant regions) early in phage infection and might similarly delay lysis, providing time for the CRISPR system to act. Further experiments would need to be done to confirm the internal mechanism of the acquired eDNA and its potential interactions with the CRISPR-Cas system. Regrettably, these will have to wait until either another medium is found to replicate these findings or Oxoid can again supply the M17 media—we suspect the activation of competence, known to be tightly and complexly regulated, during infections is the media-sensitive step.

Overall, our data link the presence of phage eDNA and CRISPR adaptation. As a natural byproduct of phage-derived lysis, the presence of phage eDNA is already associated with the threat of lysis for bacteria and its involvement in the development of CRISPR immunity shows that bacteria have multiple ways of being safely exposed to phage DNA. Moreover, this discovery brings another tool in the phage-bacteria interaction toolbox, providing another way to influence BIM generation, by modulating the levels of phage eDNA present in lysates. This can open up new avenues of research into the mechanism of CRISPR acquisition and potentially lead to the discovery of new factors influencing its activity during phage infection.

## MATERIALS AND METHODS

### Strains used and culturing

*S. thermophilus* DGCC7710 was grown in LM17 media composed of M17 powder (unless otherwise specified Oxoid, Nepean, Canada) supplemented with 5 g·L^−1^ without agitation at 37°C for overnight cultures and at 42°C for cultures used for same-day experiments (day cultures). Lysates of phages 2972 and 858 were amplified and quantified as described (20) in LM17-CaCl (LM17 media supplemented with 10 mM of CaCl_2_).

### Purification of phage particles

To purify phage particles, 0.5 M of NaCl and 10% w·v^−1^ of PEG8000 were added to 1 L of phage lysate and incubated overnight at 4°C with gentle agitation. The phages were precipitated by centrifugation at 20,000×*g* for 15 min at 4°C, and the pellet was resuspended in 5 mL of phage buffer. The solution was centrifuged at 210,000×*g* for 3 h on a CsCl gradient column formed of three 8-mL solutions of CsCl at concentrations of 0.525, 0.7,75, and 1.025 g·mL^-1^, respectively (lowest on top). Fractions of approximately 500 µL were collected starting at the indicative blue diffraction band. All fractions were tested for phage titer, and the fractions containing phage were also tested for the presence of DNA by treating a sample of each fraction with MseI and HindII (New England Biolabs, Whitby, Canada) restriction enzymes for 15 min at 37°C according to the manufacturer’s recommendations. The samples were then run on a 1% agarose gel to confirm the absence of contaminant genomic DNA (not shown).

### BIM assays

Phage lysate containing between 1e8 and 3e8 PFU*mL^−1^ was diluted to 80% original concentration with a combination of phage buffer (50 mM Tris-HCl, pH 7.5, 100 mM NaCl, 8 mM MgSO_4_), purified DNA, DNase buffer, DNase, or other supplemented enzymes or protein depending on the condition. For the MOI range assays, phage lysates were diluted with phage buffer only to the desired MOI. MOIs between 0.15 and 0.45 were used to allow for a wider range of phage titer while keeping the 80% dilution constant as all these MOIs were in the statistically significant range for the effect of purified phage treatment (Fig. 1C). For the assay, 100 µL of diluted phage and 300 µL of bacterial culture at OD_600_ between 0.4 and 0.5 as measured on a Genesys 30 Spectrophotometer (Thermo Scientific, Waltham, USA) were added to 3 mL of molten LM17-CaCl 0.75% w·v^−1^ agar and poured over LM17 1% w ·v^−1^ agar plates. The plates were incubated for 24–48 h at 42°C in sealed plastic bags before the colonies were counted. CFU counts were analyzed using the R Statistical Software (v4.2.2) (46). For most BIM assays, in order to account for the inherent variability in BIMs obtained from different bacterial cultures on different days, BIMs were reported as the log2 of the count relative to the baseline. The baseline was established for each day’s bacterial culture and phage lysate pair used by averaging the CFUs obtained from the untreated condition.

When required, single colonies were picked and used as a template for amplification by PCR using Q5 DNA polymerase (New England Biolabs, Whitby, Canada) with primers CR1_F and R as well as CR3_F and R (Table S2), according to the manufacturer’s recommendation. After verification of the size of the amplicons to confirm acquisition by gel electrophoresis, the PCR amplicons were purified using the Monarch PCR and DNA Cleanup Kit (New England Biolabs, Whitby, Canada) and sent for Sanger sequencing at the McMaster Genomics facility. The newly acquired spacers were identified by direct sequence alignment.

### DNA extraction from phage particles

Lysates were treated with 20 µg·mL^-1^ of RNase A and 2U·mL^-1^ of DNase I (New England Biolabs, Whitby, Canada) along with 10% DNase buffer 10× at 37°C for 30 min followed by heat inactivation at 75°C for 10 min. The phages were then treated with 100 µg·mL^-1^ of Proteinase K (New England Biolabs, Whitby, Canada) with SDS to a final concentration of 2% (v·v^−1^) at 37°C for 1 h. DNA was extracted using phenol:chloroform (1:1), precipitated using an isopropyl alcohol wash and ethanol, and then rehydrated in Elution Buffer (New England Biolabs, Whitby, Canada). DNA concentration was measured using a QuBit 4 Fluorometer and the QuBit dsDNA High Sensitivity Quantitation kit (Invitrogen, Waltham, USA), and DNA purity was assessed using a DS-11 series spectrophotometer (DeNovix, Wilmington, USA).

### DNA extraction from bacteria

Bacterial cultures were grown overnight and pelleted by centrifugation for 1–5 min at >12,000×*g* and resuspended in 0.8% of the original volume of 10 mM Tris-Cl pH 8.0. DNA was extracted using the Monarch Genomic DNA Purification Kit (New England Biolabs, Whitby, Canada) according to the manufacturer’s recommendations for DNA purification from Gram-positive bacteria and Archaea. DNA concentration was measured using a QuBit 4 Fluorometer and the QuBit dsDNA High Sensitivity Quantitation kit (Invitrogen, Waltham, USA), and DNA purity was assessed using a DS-11 series Spectrophotometer (DeNovix, Wilmington, USA).

### Digestion of eDNA in lysates

To fully digest eDNA from phage lysates, 256 µL of phage lysate was added to 32 µL of DNase Buffer or rCutSmart Buffer (for restriction enzymes) along with 10 µL of either DNase I, MseI, HindIII-HF, or NotI-HF (New England Biolabs, Whitby, Canada) and 22 µL of phage buffer. The mixture was incubated at 37°C for 1 h and then kept at 4°C for no more than 2 h before being used for a BIM generation assay. Controls treated with buffer only were also performed in a similar manner by replacing the volume of enzyme with phage buffer.

### Quantification of eDNA by qPCR

Quantification of eDNA was performed by qPCR using the Azure Cielo real-time PCR machine (Azure Biosystems, Dublin, USA) and primers specific to phage 858, phage 2972, and to *S. thermophilus* DGCC7710. The reaction was performed using the PowerUp SYBR Green Master Mix (Applied Biosystems, Waltham, USA) according to the manufacturer’s recommendations using the manufacturer’s standard cycling mode for primers with Tm >60°C. Standard curves were established with all three primer sets by performing qPCR on serial dilutions of a solution containing a known quantity (measured using Fluorometer and the QuBit dsDNA High Sensitivity Quantitation kit (Invitrogen, Waltham, USA) of phage 858, phage 2972, or *S. thermophilus* DGCC7710 DNA. Data analysis was performed using the R Statistical Software (v4.2.2) (46). The Ct values measured (*n* = 3) were correlated with the known concentration of DNA, and a linear regression model was established using the stats R package (v4.0.3) (46). The model was used to convert measured Ct values into DNA concentrations, and standard error was carried along using the errors R package (v0.4.0) (47).

### Liquid BIM assays and multiplex PCR

Bacterial cultures were grown to an OD600 of 0.4, and 5 mL of culture was added to 1.33 mL of phage lysate diluted to arrive at a final MOI of 0.3. CaCl_2_ was added to a final concentration of 10 mM. Control cultures were supplemented with phage buffer instead of phage lysate. The cultures were incubated overnight at 42°C, and the cells were then pelleted, and the gDNA was extracted. From 3 mL of the supernatant, eDNA was extracted using phenol:chloroform:isoamyl alcohol (25:24:1, v·v^−1^), precipitated using an isopropyl alcohol wash and ethanol and then rehydrated in Elution Buffer (New England Biolabs, Whitby, Canada).

The extracted DNA was then used as the template in a PCR reaction with a set of primers designed to target 10 spacers matching unique protospacers to 858. The primers were designed to avoid potential off-target binding to other protospacers, from either phage 2972 or phage 858. The PCR reaction was set up using Q5 polymerase (New England Biolabs, Whitby, Canada) following the manufacturer’s recommendation, using an annealing temperature of 51.4°C for CR1 and 52.7°C for CR3, chosen as the average recommended annealing temperature of all the primer pairs in the multiplex primer set. Results were visualized using agarose gel electrophoresis where the presence of a band indicates acquisition of a spacer matching one of the 10 targeted protospacers. The ability for the multiplex PCR to detect acquisition was confirmed by PCR on liquid culture infected with phage 858.

### Construction of the Δ*comEC* strain

The pCR-ComEC plasmid containing (i) the pNZ123 backbone, (ii) an artificial mini-CRISPR array composed of the CR1 leader sequence and of a single spacer targeting the *comEC* gene flanked by two repeats, and (iii) a recombination template composed of 500 bp upstream and downstream of the *comEC* gene was assembled using Gibson Assembly (New England Biolabs, Whitby, Canada) according to the manufacturer’s recommendation. The resulting plasmid was transformed into commercial NEB5α *E. coli* cells, according to the manufacturer’s recommendations (New England Biolabs, Whitby, Canada). The plasmid was then purified using the NEB Miniprep kit and then electroporated into electrocompetent *S. thermophilus* DGCC7710 made through a glycine shock protocol as described in reference 20.

Colonies of *S. thermophilus* growing on LM17 supplemented with 30 µg·μL^-1^ of chloramphenicol (Cm30) were picked and tested by PCR to show deletion of the *comEC* gene. The confirmed mutant was then serially passaged on LM17 media without antibiotics to cure the pCR-ComEC plasmid. Loss of the plasmid was assessed by a loss of resistance to chloramphenicol in liquid media by inoculating a single colony into two LM17 tubes, only one of which contained chloramphenicol.

### Statistical analyses

In order to verify statistical significance when comparing results across multiple conditions, such as fold change in BIMs or eDNA concentrations, we first used Bartlett’s test of homoscedasticity (48) to verify if the variance of the various conditions was equal. When the variances showed no significant difference and when the groups were of equal sizes, significant differences between the groups were established by a one-way analysis of variance (ANOVA) followed by Tukey’s HSD test. When the assumptions of equal variance or equal group size were rejected, Welch’s ANOVA (49) was used as an alternative, followed by the Games-Howell post-hoc pairwise comparison test (50)as suggested by recommended practices in statistics (51). Unless specified otherwise, only comparisons to the control or untreated conditions were considered. In the case of the MOI range experiments, individual pairwise tests were performed for each MOI. Statistical tests were chosen in accordance with the type of variable assessed as suggested in the handbook of biological statistics (52).

## SUPPLEMENTAL MATERIAL

Fig. S1 msphere.00453-25-s0001.eps Concentration of eDNA as a function of phage titer.

Fig. S2 msphere.00453-25-s0002.eps BIM generation with lysate treated with restriction enzymes.

Fig. S3 msphere.00453-25-s0003.eps Effect of DNase treatment on phage titer.

Fig. S4 msphere.00453-25-s0004.eps BIM generation with purified phage supplemented with phage DNA and ComS peptide.

Fig. S5 msphere.00453-25-s0005.eps Difference in effect of eDNA in S. thermophilus WT vs. Δ*comEC*.

Fig. S6 msphere.00453-25-s0006.eps BIM assay in LM17 from different brands.

Supplemental material msphere.00453-25-s0007.docx Supplemental figure legends and tables.

Raw data msphere.00453-25-s0008.xlsx Raw data for all figures.

ASM does not own the copyrights to Supplemental Material that may be linked to, or accessed through, an article. The authors have granted ASM a non-exclusive, world-wide license to publish the Supplemental Material files. Please contact the corresponding author directly for reuse.
