# Supplementary material for: CRISPR adaptation in Streptococcus thermophilus benefits from phage environmental DNA
Source: mSphere. 2025 Sep 22;10(10):e00453-25. doi: 10.1128/msphere.00453-25 (PMC12570759; doi:10.1128/msphere.00453-25)
Supplement: Supplemental material — Supplemental figure legends and tables. [file msphere.00453-25-s0007.docx]

**Supplementary Figure 1: Concentration of eDNA as a function of phage titre**Phage 2972 values are shown in green, phage 858 values in red and DGCC7710 in blue. Linear regression analysis showed no correlation between phage titre and concentration of eDNA both when considering each phage alone and when considering all lysates together. Error bars represent the standard error on each value carried over from the linear regression model established from the qPCR standard curve. Standard error was carried using the first-order Taylor series method. Two phage 858 samples were never tested with the WT DGCC7710 primers.

**Supplementary Figure 2: BIM generation with lysate treated with restriction enzymes**BIMs obtained in challenges against phage 2972. BIMs are reported as fold-change relative to the baseline, violin plots represent the probability density curve of the distribution, boxplots represent the first and third quartile of the distribution (box), the minimum and maximum (whiskers), the median (white line) as well as any outliers (black dots). The mean of each distribution is represented by an orange diamond. Significance is determined by pairwise comparison with the untreated condition using Welch’s Anova followed by Games-Howell post-hoc pairwise comparison test. The restrictions enzymes are predicted to cut the genome of phage 2972 269 times for MseI, 16 times for HindIII and 0 times for NotI.

**Supplementary Figure 3: Effect of DNase treatment on phage titer.**Titer of various phage 2972 lysates before and after DNase treatment. Each lysate was quantified using a full plate PFU assay with 3 replicates for each measurement. The DNase treatment did not significantly impact the titer of phage 2972 lysates according to the Tukey HSD test (p>0.05).

**Supplementary Figure 4: BIM generation with purified phage supplemented with phage DNA and ComS peptide**
BIMs obtained in challenges against phage 2972. BIMs are reported as absolute CFU count, violin plots represent the probability density curve of the distribution, boxplots represent the first and third quartile of the distribution (box), the minimum and maximum (whiskers), the median (white line) as well as any outliers (black dots). The mean of each distribution is represented by an orange diamond. Significance is determined by pairwise comparison with the untreated condition using Welch’s Anova followed by Games-Howell post-hoc pairwise comparison test. When supplemented, phage DNA was supplemented to a final concentration of 10 ng·μl^-1^.

**Supplementary Figure 5: Difference in effect of eDNA in *S. thermophilus* WT vs Δ*comEC***A) BIMs obtained in challenges of phage 2972 against *S. thermophilus* Δ*comEC*. BIMs are reported as fold-change relative to the baseline. B) BIMs obtained in challenges of untreated phage 2972 lysates against *S. thermophilus* WT and ΔcomEC. BIMs are reported as absolute CFU count. In all cases, violin plots represent the probability density curve of the distribution, boxplots represent the first and third quartile of the distribution (box), the minimum and maximum (whiskers), the median (white line) as well as any outliers (black dots). The mean of each distribution is represented by an orange diamond. Significance is determined by pairwise comparison with the untreated condition using Welch’s Anova followed by Games-Howell post-hoc pairwise comparison test.

**Supplementary Figure 6: BIM assay in LM17 from different brands**

A) BIMs obtained in challenges with phage 2972 lysates treated with DNase in Tekniscience and Difco brand LM17. B) BIMs obtained in challenges with phage 2972 lysates supplemented to a final concentration of 10 ng·μl^-1^ with varying DNA sources in Tekniscience and Difco brand LM17. In all cases, violin plots represent the probability density curve of the distribution, boxplots represent the first and third quartile of the distribution (box), the minimum and maximum (whiskers), the median (white line) as well as any outliers (black dots). The mean of each distribution is represented by an orange diamond. Significance is determined by pairwise comparison with the untreated condition using Welch’s Anova followed by Games-Howell post-hoc pairwise comparison test. The media brand used is indicated by a coloured bar under the X-axis.

**Supplementary Table 1: Strains and plasmids used**

| **Name** | **Source** | **Reference** |
| --- | --- | --- |
| *S. thermophilus* DGCC7710 | Félix d’Hérelle Phage Reference Center | HER 1458 |
| Brussowvirus bv2972 |  | HER 458 |
| Brussowvirus bv858 |  | HER 459 |
| pNZ123 |  | (1) |

**Supplementary Table 2 : DNA products used**

| Name | Sequence | Description |
| --- | --- | --- |
| 858qPCR_F | GCGAAGGCAGAAAAGACACC | qPCR primer for phage 858 DNA |
| 858qPCR_R | GTCAGTCGTCCGTAGTGCAA |  |
| 2972qPCR_F | AACGACATTCGGAGGGTGTG | qPCR primer for phage 2972 DNA |
| 2972qPCR_R | GATATGTCTCCCACCGCTGG |  |
| SThermoqPCR_F | ACGAGTTAAGGGCTTGACCG | qPCR primer for *S. thermophilus* DGCC7710 DNA |
| sThermoqPCR_R | CCAACTGTATCTGTCCCGGT |  |
| Cr1F | TGCTGAGACAACCTAGTCTCTC | Amplify the CR1 CRISPR array (20) |
| Cr1R | GTTGAGGCCTTGTTC |  |
| Cr3F | CTGAGATTAATAGTGCGATTACG | Amplify the CR3 CRISPR array (20) |
| Cr3R | GCTGGATATTCGTATAACATGTC |  |
| 2972SP_F | CGATGAAACGAAACTCGACACA | Amplify the supplemented amplicon single protospacer |
| 2972SP_R | TCGTTAACAACTACCGCTTTAGA |  |
| 2972_FragA_F | CATCGACCACGTTGGAAATG | Amplify the supplemented amplicon Fragment A |
| 2972_FragA_R | TCCTGAGGCTTTAGCAAGGT |  |
| 2972_ FragB_F | GGTAACGATGGTAAGGGTACGT | Amplify the supplemented amplicon Fragment B |
| 2972_ FragB_R | GAGCGGTATACATCCCACAAGA |  |
| 2972_FragC_F | AACGCTAAAGAACGAGCTCG | Amplify the supplemented amplicon Fragment C |
| 2972_ FragC _R | CTTGACACTCCGTTGGCTAT |  |
| 2972_ FragD_F | CGCTGAATGGTAACCACTCT | Amplify the supplemented amplicon Fragment D |
| 2972_ FragD_R | AAGACACTCTTAGCAGTGCC |  |
| 2972Var_F | CATCGGCAAGTGTCTACGCC | Amplify the variable region of phage 2972 |
| 2972Var_R | CGCTCTTGGTGTCTGTAGGT |  |
| 858Var_F | GTGCTATGCCAGATATTCGCTC | Amplify the variable region of phage 858 |
| 858Var_R | GGAGACACGGCAACCAAGTA |  |
| miniCRISPR | GGGTACCGAGCTCGATTCACAAGGACAGTTATTGATTTTATAATCACTATGTGGGTATAAAAACGTCAAAATTTCATTTGAGGTTTTTGTACTCTCAAGATTTAAGTAACTGTACAACAACTCCATCCATTCAAAAAGTGTGTTAAATGTTTTTGTACTCTCAAGATTTAAGTAACTGTACAACGTCGACTCTAGAGGATCCCC | Synthetic DNA product containing the mini CRISPR array with the spacer targeting the *comEC* gene. Obtained from IDT (Coralville, USA) as a minigene product. |
| GibComEC_Sp_F | CTACCGCTCGGCAAAATTGCGGGTACCGAGCTCGATTC | Gibson primer: Amplify the synthetic sequence containing the spacer targeting the *comEC* gene. |
| GibComEC_Sp_R | AGCATTTTCTGGGGATCCTCTAGAGTCG |  |
| GibComEC_US_F | GAGGATCCCCAGAAAATGCTAGCACCCATAG | Gibson primer: Amplify the upstream half of the recombination template for deletion of the *comEC* gene |
| GibComEC_US_R | TGTGGCTTAATTGTTTGGATTCTTAATTTGTCATG |  |
| GibComEC_DS_F | ATCCAAACAATTAAGCCACATAGAGGGACTC | Gibson primer: Amplify the downstream half of the recombination template for deletion of the *comEC* gene |
| GibComEC_DS_R | GCCCCGTTAGTTGAAGAAGGAGGCGTCTTCCCAGTCAAAG |  |
| pNZ123_Lin_F | CCTTCTTCAACTAACGGGGCAGGTTAG | Primers, linearizes the pNZ123 plasmid |
| pNZ123_Lin_R | GCAATTTTGCCGAGCGGTAGC |  |
| ΔcomEC_confirm_F | ATGAGCCTGCTTCAATACGG | Primers, amplifies across the *comEC* gene (or deletion) in both WT DGCC7710 and ΔcomEC mutant |
| ΔcomEC_confirm_R | TATCGTGAAAGTCATGGCGG |  |
| 858_CR1UM_Rev1 | TCGTCAACCCTTTAAAAGCCA | Reverse multiplex primers binding to CR1 spacers matching protospacers unique to phage 858. |
| 858_CR1UM_Rev2 | CCACCACAAAAACCTTGATGGG |  |
| 858_CR1UM_Rev4 | ACCGTTTAGGCTTGCAGAAA |  |
| 858_CR1UM_Rev5 | AGTGGGTTATAAGATGTTGCAATTGT |  |
| 858_CR1UM_Rev6 | ACATGCTAGACGGATTTCTCG |  |
| 858_CR1UM_Rev7 | TCTGTTGAATGTGTTGTGGTATTT |  |
| 858_CR1UM_Rev8 | TGCTTTCAAAAGTTCTGTCAAGCT |  |
| 858_CR1UM_Rev9 | AGTGTAATGGTTTGCAATTTTAGCT |  |
| 858_CR1UM_Rev10 | AGGAGCTTCAGCAATCGTATCA |  |
| 858_CR1UM_Rev11 | TCGCTGAATATGGTGCCAACT |  |
| 858_CR3UM_Rev1 | AGAGCGTATTCCTTTACCTGCA | Reverse multiplex primers binding to CR3 spacers matching protospacers unique to phage 858. |
| 858_CR3UM_Rev2 | TCCTCCTTGCACCATTCTTGA |  |
| 858_CR3UM_Rev3 | GCACCATTCTTGATACAACCACC |  |
| 858_CR3UM_Rev4 | ACAACCACCACAAAAACCTTGA |  |
| 858_CR3UM_Rev5 | CCTTGATGGGAGTCGAGTGG |  |
| 858_CR3UM_Rev6 | ACCGTCTTACCCTCTATGATAGAC |  |
| 858_CR3UM_Rev7 | ACAAACCAGATTGATTTTCTGTAAGA |  |
| 858_CR3UM_Rev8 | TGCAAAATTACAAGAAAAATCTTCCCG |  |
| 858_CR3UM_Rev9 | ACCATGCTTTCAAAAGTTCTGTCA |  |
| 858_CR3UM_Rev10 | TAGCCAGTGCATTGTTCCGT |  |
| CR1_UM_For | ACAACCTAGTCTCTCACTTGTTGA | Forward primer for all multiplex CR1 primers |
| CR3_UM_For | GGCAAGACCTGGTCCACATA | Forward primer for all multiplex CR3 primers |

REFERENCE:

1. De Vos W. 1987. Gene cloning and expression in lactic streptococci. FEMS Microbiol Lett 46:281–295. https://doi.org/10.1016/0378-1097(87)90113-3
